# Fertility in congenital adrenal hyperplasia due to 21-hydroxylase deficiency: a review

**DOI:** 10.3389/fendo.2025.1682341

**Published:** 2025-11-28

**Authors:** Zuzanna Roszkowska, Małgorzata Bobrowicz, Joanna Betlejewska, Joanna Hubska, Beata Rak-Makowska, Urszula Ambroziak

**Affiliations:** 1 Student Scientific Club “Endocrinus” Affiliated to the Department of Internal Medicine and Endocrinology, Medical University of Warsaw, Warsaw, Poland; 2 Department of Internal Medicine and Endocrinology, Medical University of Warsaw, Warsaw, Poland; 3 Doctoral School of the Medical University of Warsaw, Warsaw, Poland; 4 Laboratory of Experimental Medicine, Medical University of Warsaw, Warsaw, Poland; 5 Centre of Postgraduate Medical Education, Centre of Translational Research, Department of Biochemistry and Molecular Biology, Warsaw, Poland

**Keywords:** congenital adrenal hyperplasia, fertility, sexuality, adrenals, 21-hydroxylase

## Abstract

Congenital adrenal hyperplasia (CAH) due to 21-hydroxylase deficiency is a rare autosomal recessive disorder affecting adrenal steroidogenesis, leading to cortisol deficiency, androgen oversecretion and adrenal glands’ hyperplasia. While neonatal screening programs facilitate early diagnosis and treatment, CAH remains a complex condition with significant implications for fertility in both men and women. Women with CAH may experience menstrual irregularities, anovulation, prenatal virilization and psychological problems, while men face the problem of testicular adrenal rest tumors (TARTs), which can impair spermatogenesis, as well as experience sexual dysfunctions. Despite advances in the management of CAH, the issues of fertility and quality of sexual life still pose a challenge in this group of patients. This review aims to present the topic of fertility in CAH, taking into consideration the latest research and novel treatment options and underscores the importance of neonatal screening programs as well as personalized, team-based management to improve both reproductive outcomes and quality of life in CAH patients.

## 1 Introduction

Congenital adrenal hyperplasia (CAH) is a rare autosomal recessive disorder caused by enzyme deficiencies in adrenal steroidogenesis, which leads to decreased cortisol production and oversecretion of adrenocorticotropic hormone (ACTH) (1). As a result, ACTH excess, both stimulates overproduction of adrenal androgens and causes adrenal hyperplasia. More than 95% of CAH is caused by decreased activity of 21-hydroxylase (21-OH) due to mutations in the 21-hydroxylase (*CYP21A2*) gene (2, 3), thus this form will be addressed in our review. The clinical phenotype encompasses: the classic form including severe salt-wasting (SW) form with 0% to 1% residual enzyme activity, simple virilizing (SV) form with approximately 2% residual enzyme activity, and the mild nonclassic (NC-CAH) form with around 20% normal enzyme activity (4), known also as late onset CAH. The incidence of the classic type ranges from 1:10,000 to 1:20,000, depending on the ethnic background, whereas the nonclassic form is more common occurring in 1:200-1:2000 live births (3, 5, 6).

SW CAH is a life-threatening condition due to risks of severe hyponatremia, hyperkalemia, hypotension, and adrenal crisis if untreated. In case of SV and SW form, females may present with ambiguous genitalia, ranging from clitoromegaly to nearly male-like features (7). Some cases can be suspected via prenatal ultrasound, while other, mild cases may remain unnoticed until clitoromegaly progresses (8). Males have adequate image of external genitalia; the only feature may be genital hyperpigmentation. In fact, CAH is the most common among the 46,XX differences of sex development (DSD) (1). Severely virilized females can be misassigned as males at birth, resulting in irreversible masculinizing surgeries, such as hypospadias repair or scrotoplasty, which may not align with their chromosomal sex or future gender identity (1). Early and accurate diagnosis is essential to ensure appropriate sex assignment (8).​ However, decisions regarding sex of rearing in severe 46, XX CAH remain complex and must be made through a multidisciplinary approach, taking into account surgical outcomes, psychosocial development, and potential future fertility. While some experts support female assignment due to the potential for fertility with appropriate treatment, others consider male assignment in cases of markedly masculinized genitalia, emphasizing individual variability and long-term psychosocial outcomes (9, 10). NC-CAH, presents with milder cortisol deficiency, genitalia are usually non affected, and the condition may manifest solely as premature pubarche, hirsutism, acne, menstrual irregularities, chronic anovulation, and/or infertility. Due to unspecific symptoms or being asymptomatic they may remain undiagnosed until adulthood (11–13).

**Figure 1:**
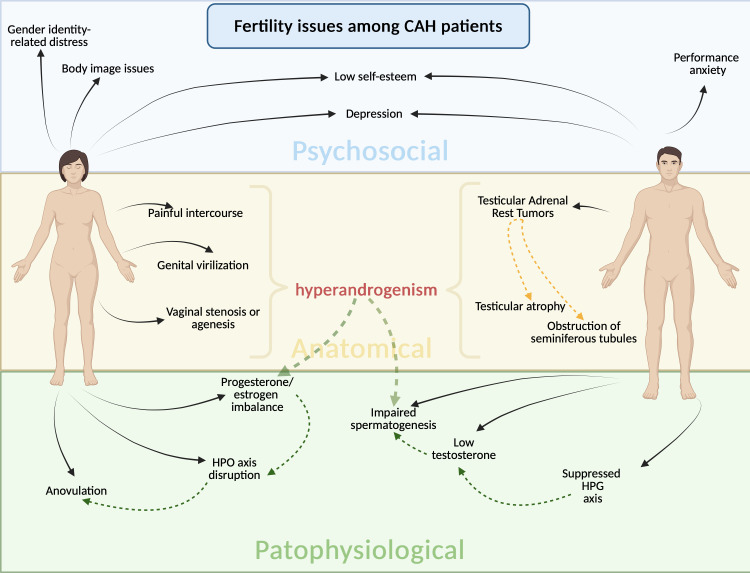
Fertility issues in patients with congenital adrenal hyperplasia, categorized into pathophysiological, anatomical, and psychosocial domains, with sex-specific distinctions. In female patients, physiological factors include disruption of the hypothalamic–pituitary–ovarian axis, anovulation, and progesterone/estrogen imbalance. Anatomical challenges include genital virilization, vaginal stenosis or agenesis, and dyspareunia (painful intercourse). In male patients’ pathophysiological factors include suppression of the hypothalamic–pituitary–gonadal axis, low testosterone levels, and impaired spermatogenesis. Anatomical factors involve testicular adrenal rest tumors/tissue, testicular atrophy, and obstruction of seminiferous tubules. Across both sexes, psychosocial factors such as low self-esteem and depression, which can further contribute to fertility difficulties. The figure emphasizes the complex and intersecting biological and psychological challenges faced by CAH patients in achieving fertility.

The severity of symptoms of classic CAH requires immediate and proper diagnosis, therefore the neonatal screening program was introduced in over 50 countries, including Poland since 2016 (8). The incidence in the Polish population is approximately 1 in 15:000 live births (14), which aligns with the data from other western countries. Patients with CAH require lifelong glucocorticoid (GC) replacement therapy, whereas mineralocorticoid (MC) supplementation is necessary within the classic SW form of this disease. Effective suppression of adrenal androgens usually requires supraphysiological doses of glucocorticoids. Managing CAH remains challenging, especially when trying to conceive, as well as during the pregnancy and prenatal period, with distinct implications for men and women. Excessive secretion of adrenal hormones may disrupt the hypothalamus-pituitary-gonadal axis resulting in menstrual irregularities, anovulation, and ovarian dysfunction in females, while in males, they may lead to low testosterone levels, reduced sperm counts, and testicular adrenal rest tumors (TARTs). Moreover, it can lead to atypical genitalia, further complicating reproductive health (**Figure 1**). These disturbances impair both fecundity (the biological capacity to reproduce) and fertility (the actual ability to achieve pregnancy). In the recent years many extensive reviews on the topic have been published presenting the key aspects of CAH management (1, 8).

## 2 Female fertility

One of the first studies on fertility in 21-OHD conducted in 1999, found that patients with classic form were less likely to seek motherhood and had fewer pregnancies compared to healthy control (15). However, subsequent research has yielded inconclusive results (16). On one hand, Swedish population-based study involving 272 women with 21-OHD reported a lower pregnancy rate, with only 25.4% of 21-OHD with offspring compared to 45.8% of controls (16). Interestingly, this difference in motherhood rate was observed only in women with SW CAH, while those with SV and NC-CAH had pregnancy rates comparable to controls (16). Conversely, Auer et al., in their multicenter study on 72 21-OHD patients (NC = 34, SV = 21, SW = 17) demonstrated favorable pregnancy outcomes, however pointed out a prolonged time to conceive in this population (17). Likewise, a UK population-based study by Casteras et al., found that the pregnancy rate among women with classic CAH was similar to the one in the general UK population. However, women with 21-OHD were less likely to pursue motherhood, as only 23.6% of 106 CAH women with classic forms of CAH expressed a desire to become a mother (18). All together, these findings suggest that normal fecundity may be achieved in CAH affected women, at least in the less severe forms.

Infertility may be an issue in NC-CAH, a condition that is often misdiagnosed as polycystic ovary syndrome (PCOS). In fact, a study by Trakakis et al. revealed that infertility was the primary symptom leading to diagnosis in 13% of NC-CAH cases (19, 20). However, according to studies presented below, only 10 to 30% of NC-CAH women has experienced infertility (21, 22). An international multicenter retrospective/prospective study conducted on a group of 109 NC-CAH women showed that 68% pregnancies occurred even before the NC-CAH had been diagnosed, indicating the possibility of spontaneous pregnancy without any treatment (21). Similarly, a retrospective studies by Bidet et al. on a group of 190 women with NC-CAH, and by Eyal et al. on a 75 NC-CAH patients reported infertility rates comparable to general population, making the problem of subfertility in NC-CAH women relative (22, 23). Additionally, these studies suggest that women with NC-CAH without GC treatment experience a higher frequency of miscarriage compared to the general population, which is consistent with findings from other studies. This appears to be largely due to elevated androgen levels, which may interfere with endometrial receptivity, as well as hormonal imbalances such as increased progesterone levels during the follicular phase. Importantly, early initiation of glucocorticoid therapy has been shown to markedly improve pregnancy outcomes in these patients (21–23). Additional studies in this area are summarized in **Table 1**.

**Table 1:** Summary of key studies evaluating reproductive intentions, fertility outcomes, and birth rates among women with classical and nonclassic congenital adrenal hyperplasia.

Study	Country	Sample	Focus	Main findings
What causes low rates of child-bearing in congenital adrenal hyperplasia?(Meyer-Bahlburg, H. F) (15)	USA, 1999	N not specified; women with classical CAH	Reproductive intentions and outcomes	Less than 30% considered motherhood
Reproductive Outcome of Women with 21-Hydroxylase-Deficient Nonclassic Adrenal Hyperplasia (Moran, C. et al.) (21)	USA/Mexico, 2006	101 women with nonclassical CAH	Spontaneous fertility and diagnosis timing	68% pregnancies before NC-CAH diagnosis
Reassessing fecundity in women with classical congenital adrenal hyperplasia (CAH): normal pregnancy rate but reduced fertility rate (Casteràs, A., De Silva, P., Rumsby, G. & Conway, G. S.) (18)	UK, 2009	106 women with classical CAH	Motherhood intentions and pregnancy outcomes	90% pregnancy success among those attempting; overall rates similar to general population; 23.6% women considered motherhood
Fertility in Women with Nonclassical Congenital Adrenal Hyperplasia due to 21-Hydroxylase Deficiency (Bidet, M. et al.) (22)	France, 2010	190 women with nonclassical CAH	Fertility outcomes and miscarriage incidence	Fertility comparable to general population; miscarriage higher without treatment
Fertility outcome and information on fertility issues in individuals with different forms of disorders of sex development: findings from the dsd-LIFE study (Słowikowska-Hilczer, J. et al) (24)	Multiple European countries, 2017	221 women with classical and nonclassical CAH	Childbirth prevalence and ART use	14.7% women had children; 1.9% conceived via ART
Pregnancy in women with nonclassic congenital adrenal hyperplasia: Time to conceive and outcome(Eyal, O. et al) (23)	Israel, 2017	75 women with nonclassical CAH	Fertility and miscarriage rates	Fertility similar to general population; miscarriage higher without treatment
Reproductive and Perinatal Outcomes in Women with Congenital Adrenal Hyperplasia: A Population-based Cohort Study (Hirschberg, A. L. et al.) (16)	Sweden, 2021	272 women with classical CAH	Pregnancy rates and genotype severity	50% reduced pregnancy frequency; conception success linked to genotype severity

The table presents findings from multiple international studies, comparing pregnancy rates, childbearing prevalence, and the impact of treatment.

Several factors have been proposed to explain the lower fertility rate (i.e. live births per woman) in 21-OHD patients in comparison to general population. Firstly, the most apparent factor is adrenal steroid excess, which is particularly pronounced in poorly controlled patients. A study by Bachelot et al. (25) showed that women with 21-OHD with optimized GC therapy resulting in normal androgen and progesterone levels, exhibited normal LH pulsatility. Conversely, in patients with poor disease control, persistent excess of adrenal steroids was associated with a reduction in the frequency and amplitude of LH pulses, likely due to excessive progesterone secretion. Elevated progesterone levels during the follicular phase, even with appropriate GC supplementation, have been associated with amenorrhea and infertility in individuals with 21-OHD (25). Increased progesterone concentrations can disrupt the GnRH pulse generator, ultimately interfering with ovulation and then with embryo implantation due to endometrial thinning, and hindering sperm migration by causing the cervical mucus to become thick and less permeable (26). These findings suggest that increased androgen levels promote early follicle development but subsequently hamper follicle growth in later stages (27) Recently, there has been growing interest in 11-oxygenated (11-oxo) androgens, as they have emerged as a novel source of androgens (28–30). 11-oxo androgens are adrenal-derived C19 steroids, including metabolites such as 11β-hydroxyandrostenedione (11OHA4) and 11β-hydroxytestosterone, which are further converted in peripheral tissues to potent androgens like 11-ketoandrostenedione and 11-ketotestosterone (11KT) (31). These steroids significantly contribute to the overall androgen pool in women, since they may increase total androgenic activity beyond what is reflected by traditional measurements of testosterone and DHEA (31). A study conducted by Auer et al. demonstrated that 11-oxo androgens, particularly 11KT and 11OHA4, were significantly elevated in CAH female patients experiencing menstrual disturbances, including amenorrhea. These findings indicate that 11-oxo androgens may play a role in hypothalamic-pituitary-gonadal axis regulation, contributing to reproductive dysfunction in women with CAH (32). Moreover, recent findings indicate that the 11-oxo androgens can be biomarkers of androgen excess in 21-OHD, as they are higher in women with chronic anovulation compared to control group of healthy women. Similarly, women with untreated nonclassic 21-OHD also show elevated 11-oxyandrogen levels compared with age- and BMI-matched healthy controls (33, 34). Due to the challenging measurement process, 11-oxyandrogens are not yet widely implemented in clinical practice. Unfortunately at present, the assessment of 11-oxyandrogens remains more of an emerging research trend rather than a standard diagnostic tool (35). However, efforts are currently underway to improve their clinical accessibility by standardizing LC–MS/MS assays, establishing reference ranges, and integrating 11-oxyandrogens into broader steroid profiling panels. Secondly, other factors potentially contributing to the reduced fertility in women with congenital adrenal hyperplasia are long-term consequences of genital surgery interventions performed in childhood in order to adjust virilized external genitalia, enable normal urinary and sexual function. However, such procedures may have lasting effects on sexual function, including a reduced vaginal introitus, decreased vaginal lubrication, dyspareunia, diminished clitoral sensitivity (as a consequence of surgical intervention), and overall reduced sexual satisfaction (2). Nevertheless, surgical techniques have advanced considerably over time. Modern procedures prioritize the preservation of innervation and clitoral sensation to maintain erotic sensitivity and orgasmic capacity following clitoroplasty and vaginoplasty techniques have been refined to achieve better functional and anatomical outcomes (36).

Studies have shown that women with CAH statistically often experience sexual debut later than the healthy control group or may not experience it at all, and regarding males with CAH, there is a lack of data on the age of sexual debut (37). Additionally, they report significantly less satisfying sexual functioning, such as desire, arousal, lubrication, and pain, especially in those at Prader IV-V stages (38–40). A French study including 35 patients with CAH, showed that in CAH women with Prader stage I-III (n=22), the sexual satisfaction was comparable to a control group of healthy women (n=69) (39).

In addition, psychosexual factors also contribute to subfertility in women with CAH. A study by Meyer-Bahlburg et al., found that while most women with CAH identified as heterosexual, the prevalence of bisexual and homosexual orientation was higher not only among those with classical CAH but also in those with NC-CAH. Our own research indicates increased prevalence of homosexual, as well as bisexual orientation among 21-OHD women compared to healthy control groups (41). In another study this outcome was linked to the extent of prenatal androgen exposure (37). Bisexual or homosexual orientation showed a moderate correlation with overall measures of masculinized non-sexual behavior and was independently predicted by both prenatal androgen exposure and childhood behavioral masculinization (42). Women with classical CAH may face stigma in romantic and sexual relationships, related to both their physical appearance and genital differences. A study which included 62 of classic CAH women (aged 18–51 years) showed that 40% of them report feelings of distress, and/or embarrassment (43). Some studies report on patients preferring to avoid sexual relationships entirely due to CAH diagnosis, leading to challenges in forming long-term romantic partnerships (43, 44). Internalized stigma further contributes to low self-esteem and feelings of abnormality, which may be associated with higher rates of psychiatric conditions such as depression (43). Additionally, according to multicenter study, conducted by Tschaidse et al., on a group of 203 classic CAH and NC-CAH, women with CAH report poorer body image and lower physical, psychological, and social quality of life compared to a healthy population (45). Chronic glucocorticoid therapy has been linked to anxiety, sleep disturbance, and mood/cognitive symptoms in CAH—effects that are most evident with supraphysiologic exposure and appear more frequent with long-acting agents (prednisolone/dexamethasone) than with hydrocortisone; nonetheless, some cohorts report poorer mental-health/vitality even without clear overtreatment (46–49).

## 3 Managing female infertility

The primary approach to managing infertility in women with CAH is to optimize glucocorticoid therapy to promote regular ovulation and menstruation (50). Increasing the GC dose may be beneficial to establish an ovulatory cycle and create an endometrial environment that is conducive to conception (51). The preferred GC for CAH treatment is hydrocortisone given three times daily due to its short half-life and additional mineralocorticoid effect. However, it may be necessary to increase the GC dose slightly, either by adding an additional dose, switching to a longer-acting GC such as prednisone or prednisolone, although usage of dexamethasone is still debatable (3, 50, 51). The idea behind the use of longer-acting preparations is their ability to suppress stronger ACTH and lower progesterone levels, facilitating pregnancy (3). It is suggested that optimal hormones level for achieving normal ovulatory cycles and fertility should be maintained under 8–10 ng/mL for 17-hydroxyprogesterone (17-OHP), and androgen concentrations—particularly testosterone and androstenedione—should remain within the normal female reference range, preferably in the lower half (52). Progesterone level allowing implantation should be below 0,63 ng/mL, Moreover, GC therapy should be considered in hyperandrogenic women with NC-CAH who struggle to conceive naturally and exhibit overt or subclinical ovulatory dysfunction, as it has been shown to improve fertility and prevent recurrent miscarriages (53). In particular, short-term GC treatment, among NC-CAH women who had previously failed to conceive without GC treatment, has been demonstrated to significantly reduce adrenal androgen levels, improve ovulatory function and endometrial receptivity, and consequently shorten the time to conception from an average of approximately 12 months to fewer than 6 months (23). Interestingly, study conducted by Krysiak et al. indicates that treatment with metformin in patients with NC-CAH and concomitant type 2 diabetes mellitus (DM) may help reduce 17-OHP and androgen levels, particularly in women with untreated DM before (54). These effects may indirectly enhance ovulatory function and support fertility in this subgroup, yet larger studies are needed to validate its efficacy. Hydrocortisone, prednisone, and prednisolone are inactivated by placental 11β-hydroxysteroid dehydrogenase type 2 (11β-HSD2) and therefore have no effect on the fetus. The use of dexamethasone is generally contraindicated due to its ability to cross the placenta and affect the fetus, therefore its administration should be most often stopped when the conception is achieved, or according to some authors even before (3). Potential adverse effects of supraphysiological doses of GC include hypertension, diabetes, metabolic dysfunction-associated steatohepatitis, osteoporosis, glomerulosclerosis, or depression (55). Importantly, the administration of dexamethasone carries the greatest metabolic burden, as its long half-life and strong glucocorticoid potency can lead to prolonged suppression of the hypothalamic-pituitary-adrenal (HPA) axis and a higher risk of iatrogenic Cushing’s syndrome, insulin resistance, and adverse cardiovascular outcomes (56).

In patients whose ovulation fails to resume despite adequate glucocorticoid and mineralocorticoid therapy with satisfactory suppression of androgens and progesterone, ovulation can be induced with injectable gonadotropins or clomiphene (57, 58). Optimalization of mineralocorticoid therapy in SW and SV forms, may contribute to improved reproductive function through better control of water-electrolyte homeostasis and suppression of excessive androgen production, although direct clinical evidence supporting the elimination of the need for gonadotropin therapy remains limited (7, 8, 20). Although the precise mechanism remains unclear, it is hypothesized that improved volume status and electrolyte balance may enhance ovarian perfusion and endometrial receptivity (8). *In vitro* fertilization (IVF) is another option (59). Unfortunately, there is limited data on the efficacy of IVF, as CAH is relatively rare, and the studies on this topic are scarce. In rare cases, bilateral adrenalectomy has been performed in women with persistent severe hyperandrogenism despite high-dose glucocorticoid therapy (60), although this approach is not recommended by the Endocrine Society Clinical Practice Guidelines (2, 3). Case reports have described the restoration of regular menstrual cycles or even spontaneous pregnancies following the procedure. A meta-analysis of 48 cases across 32 studies reported that three of the 35 women undergoing adrenalectomy for primary infertility later conceived, resulting in six healthy live births (60). Additionally, three patients operated on for obesity and virilization experienced resumption of spontaneous menstruation after surgery (61). However, bilateral adrenalectomy carries a significant risk of adrenal crisis and may lead to the development of adrenal rest tissue, potentially resulting in persistent hyperandrogenism (62). Modified-release hydrocortisone (MR-HC) is emerging as a superior therapeutic option compared to conventional glucocorticoid therapy in patients CAH. MR-HC mimics the natural circadian rhythm of cortisol, preventing early morning androgen excess, and stabilizes cortisol levels throughout the day. A randomized, controlled trial conducted by Merke et al. demonstrated that MR-HC improved disease outcomes based on biochemical findings (17-OHP levels) (63–67). Additionally, recent findings showed that MR-HC leads to significant reductions not only in traditional markers like 17-OHP and androstenedione, but also in 11-oxosteroids, including 11OHA4 and 11KT (63, 68). After further clinical studies, it may be worth considering this treatment for individuals seeking pregnancy. In contrast to traditional glucocorticoid therapy which often leads to peaks and troughs in cortisol levels and can result in both under- and overtreatment, modified-release hydrocortisone provides a more physiologic cortisol profile, offering improved hormonal control, reduced androgen excess, and potentially better reproductive and metabolic outcomes (69–71). Importantly, currently some novel treatment modalities are emerging including corticotropin-releasing factor 1 (CRF1) antagnosists – crinecerfont and tildacerfont, anti-ACTH monoclonal antibody and melanocortin receptor type 2 (MC2R) antagonist atumelnant (reviewed in (72)). Currently, the most advanced agent in clinical trials in crinecerfont. Although no data are currently available on pregnancy outcomes in patients with CAH treated with this agent clinical studies have demonstrated that crinecerfont significantly improved biochemical control by reducing ACTH and androgen levels. This hormonal stabilization may be associated with a potentially favorable impact on reproductive function, although further research is needed to confirm this (73, 74).

Before attempting pregnancy, it is crucial to investigate the genetic status of the partner. If the partner is not a carrier of mutant genes resulting in CAH, all offspring will be carriers. However, if the partner is a carrier, there is a 50% risk of the offspring having CAH. Based on the incidence of classic CAH, approximately 1 in 60 individuals carry the allele responsible for classic CAH (20). Although heterozygous carriers of CYP21A2 mutations are generally considered clinically unaffected, some may exhibit subtle signs of hyperandrogenism such as acne or menstrual irregularities, particularly in the context of mild or compound heterozygous mutations (75). While ACTH stimulation testing may reveal a modest rise in 17-OHP levels, genetic testing remains the gold standard for carrier identification (8). The prevalence of classic CAH due to 21-hydroxylase deficiency in the general population ranges from 1:10, 000 to 1:15, 000, according to newborn screening data. Meanwhile, the prevalence of NC-CAH is higher, at approximately 1:1000, but it is significantly more common in certain populations, including Ashkenazi Jews (up to 1:27), Hispanics, Southern Europeans, and Middle Eastern populations, with estimated prevalence ranging from 1:200 to 1:500 (75). The overall risk of being a carrier, estimated using the Hardy-Weinberg equilibrium based on newborn screening data, is about 1:55 (51). Carriers of CAH are usually unaffected but can pass the affected gene to their offspring. While carriers may exhibit a slight increase in 17OHP, this is not a reliable indicator, and genetic testing is crucial to confirm carrier status. Carriers do not exhibit the phenotypic features characteristic of CAH, making the condition difficult to suspect (76). If the carrier status of the partner of a CAH patient is unknown, the risk of giving birth to a child with classic CAH is approximately 1:120 and the risk of giving birth to a child with nonclassic CAH is 1:360 (77, 78). If both parents are carriers of genes mutations responsible for CAH, the risk of having an affected child is 25%, 50% of children will be carriers themselves, and 25% of children will be unaffected. Routine genetic carrier screening for 21-OHD is becoming increasingly affordable and is recommended by the American College of Obstetrics and Gynecology to be offered to all pregnant patients, those seeking to become pregnant during preconception visits and the partners of CAH patients (20, 79).

Although, many CAH women achieve pregnancy with proper multidisciplinary medical care, it is important to underline risk of obstetric complications. Studies have indicated an increased risk of gestational diabetes mellitus (GDM) among women with CAH, potentially due to factors such as obesity, insulin resistance, and glucocorticoid therapy. A systematic review reported a relative risk of 2.67 (95% confidence interval 1.29–5.12) for GDM in CAH patients compared to the general population (80). Existing data from retrospective studies regarding gestational hypertension or preeclampsia in this population are limited and inconsistent, highlighting the need for further prospective research. The meta-analysis showed that approximately 67.8% of CAH patients underwent cesarean delivery, which is 3.86 times higher than the rate in the general population (80). Proper management of glucocorticoid therapy during labor and delivery is crucial to prevent adrenal crises. Stress-dose steroids should be administered during labor and the immediate postpartum period to ensure adequate hormonal coverage (80, 81).

## 4 CAH male fertility

A Swedish population-based national cohort study, involving 30 males (aged 19–67 years), showed that male patients with classic form 21-OHD were less likely to become a father (82). According to that study conducted by Falhammar et al. reported a fertility rate of 0.9 ± 1.3 children per father among adult males with classic 21-OHD, which is significantly lower compared to the general Swedish male population, which has an average of 1.8 ± 0.5 children per father (82). Additional studies in this area are summarized in **Table 2**. The impact of inadequate androgen regulation on various aspects of sexual function in men with CAH, including libido and erectile function, remains an underexplored area of research. In a study by Dudzińska et al. involving 20 adult men with CAH due to 21-OHD, testosterone concentrations were within the low-normal reference range in the majority of participants (83), which was attributed to suppressed gonadotropin secretion resulting from increased aromatization of adrenal-derived androgen precursors to estrogens (75). These estrogens exert negative feedback on the HPG axis, further dampening the production of LH and FSH. Additionally, the presence of TARTs—which produce steroids independently of gonadotropin regulation—may contribute to further suppression of endogenous gonadotropins (84). The terminology regarding TARTs remains a matter of discussion: while some authors refer to them as “tumors” due to their potential to form compressive masses, others prefer the term “tissue” to emphasize their benign, non-neoplastic nature (1). This distinction highlights the importance of understanding TARTs as a functional steroidogenic residue rather than a true neoplastic process.

**Table 2:** Summary of key studies evaluating fertility and reproductive outcomes in men with congenital adrenal hyperplasia (CAH).

Study	Country	Sample	Study focus	Main findings
Pituitary gonadal axis and child rate in males with classical 21-hydroxylase deficiency (Jääskeläinen, J., Kiekara, O., Hippeläinen, M. & Voutilainen, R.) (70)	Finland, 2000	29 men with classic CAH	Comparison of fertility rates vs. general population	Fertility rate significantly reduced (child rate: 0.07 vs. 0.34); oligozoospermia frequent
Fertility, sexuality and testicular adrenal rest tumors in adult males with congenital adrenal hyperplasia (Falhammar, H. et al.) (67)	Sweden, 2012	30 adult men with classic CAH	Fertility status, TART prevalence, sexuality	33% had biological children; high prevalence (86%) of TART; reduced fertility
High prevalence of reduced fecundity in men with CAH (Reisch, N. et al) (71)	Germany, 2009	22 adult men with classic CAH	Paternity and hormonal control	23% fathered children; poor hormonal control linked to infertility
Health Status of Adults with Congenital Adrenal Hyperplasia: A Cohort Study of 203 Patients (Arlt, W. et al.) (72)	UK, 2010	65 adults with CAH (majority classic, some nonclassic)	Fertility attempts, success, treatment	only 37% attempted conception; 67% had been successful (after spontaneous conception or fertility treatment)
Clinical Outcome, Hormonal Status, Gonadotrope Axis, and Testicular Function in 219 Adult Men Born With Classic 21-Hydroxylase Deficiency. A French National Survey (Bouvattier, C. et al.*)* (73)	France, 2015	219 men with classic CAH (registry-based)	National evaluation of fertility and IVF usage	24% had biological children; 11% via assisted reproduction (mainly IVF)
Reduced Frequency of Biological and Increased Frequency of Adopted Children in Males With 21-Hydroxylase Deficiency: A Swedish Population-Based National Cohort Study (Falhammar, H. et al.) (74)	Sweden, 2017	221 men with CAH and 22,100 controls	Impact of neonatal screening on fertility	Reduced fertility in men born before screening; normal fertility in screened classic and nonclassic CAH

The table presents findings from cohort and population-based studies assessing paternity rates, childbearing outcomes, and the impact of clinical factors such as neonatal screening and fertility treatment.

The main reason of fertility issues in men are TARTs, which are benign testicular lesions, usually found in patients with CAH. Due to volume and pressure on the testis, TART can obstruct seminal ducts, as well as be a cause of atrophy of Sertoli and Leydig cells. All the factors mentioned above may impair testicular function. The prevalence of TARTs ranges from 14% to 86%, more often among younger patients (85). TARTs, a major contributor to gonadal dysfunction in CAH (86), were detected in 94% of patients in the study by Stikkelbroeck et al., and highlighting their high prevalence (76). These tumors are thought to originate from ectopic adrenal cells that migrate and become embedded in the testes during embryogenesis, and proliferate due to elevated serum ACTH concentration (77), Although some studies point to other origins, like fetal Leydig cells or adult-Leydig-cell precursors (88). It has been demonstrated that TART development is favored by poor hormonal control, although TARTs also occur in well-controlled patient (89). The formation of TARTs is particularly concerning during childhood and puberty, as these developmental stages appear to be critical periods for their emergence (84). TARTs in individuals with CAH typically remain stable during adulthood; however, a reduction in tumor size has been observed with specific therapeutic interventions, such as adrenostatic agents (e.g., metyrapone) or higher-dose dexamethasone therapy (84). Given the potential impact of TART on gonadal dysfunction, regular monitoring is strongly advised, with evaluations recommended at intervals of approximately three to five years to facilitate early detection and appropriate management (1). Surgical removal of TARTs has not been proven to restore testicular function and is therefore generally reserved for cases where tumor size causes significant pain or discomfort. In patients with detectable TARTs, early consideration of sperm cryopreservation may be advisable to preserve future fertility options (84, 87).

Although men with CAH may exhibit normal or near-normal serum testosterone concentrations due to adrenal androgen production, this does not necessarily reflect preserved testicular function. Clinically low testosterone levels are still observed in a subset of patients (90). These mechanisms, especially when combined, can severely affect not only men’s fertility, but also their overall sexual well-being (83). Importantly, glucocorticoid overtreatment aimed at controlling androgen excess can paradoxically suppress testicular function and exacerbate hypogonadism; however, this effect is typically reversible when glucocorticoid regimens are optimized (91). A prospective study in males with CAH showed that switching from standard GC treatment to MR-HC resulted in a significant reduction in 17-OHP concentrations and a notable improvement in semen parameters, including a significant increase in total sperm counts after 12 months of treatment (median rise from 20.8 million to 44.3 million) (92).

Emerging data also point to a role of other adrenal steroids and metabolites, particularly progesterone and 11-oxygenated androgens such as 11OHA4 and 11-KT, in influencing male reproductive function (93). These 11-oxo androgens are biologically active yet remain outside the regulatory influence of gonadotropins. Their accumulation may adversely affect HPG axis regulation and sexual health, even among men whose serum testosterone appears within normal limits. Moreover, their potential to alter sexual behavior and fertility underscores the complexity of androgen signaling in CAH and highlights the need for individualized hormonal assessment beyond conventional testosterone measurements (32).

When planning for fatherhood, men with CAH may need adjustments to their medical therapy to enhance fertility outcomes. Infertility and TARTs require GC treatment intensification including incorporation of dexamethasone, which can suppress ACTH levels, potentially reducing TART size and improving spermatogenesis. Regarding NC-CAH, which is generally milder and often asymptomatic, its relevance in fertility counseling becomes apparent when clinical symptoms of hypogonadism or TART are detected, which is rare (51, 94, 95). Due to their rarity, TARTs clinical relevance NC-CAH group remains limited. As a result, routine scrotal ultrasound screening is not generally recommended for asymptomatic males with NC-CAH (84, 90).

## 5 Psychological aspects of fertility

In the context of sexuality and fertility of CAH patients, psychological factors play a crucial role. There are studies suggesting that prenatal exposure to androgens can affect brain development and behavior in adulthood (96). In some cases, women exhibit masculine traits in their behavior (referred to as “tomboy”), but they still lead a predominantly feminine lifestyle (44, 44, 97). Virilization can affect body image and intimate relationships, with up to 41% reporting that CAH complicates forming romantic partnerships (98). As outlined above (see Paragraph 2), patients with moderate–to–severe genital virilization may undergo feminizing procedures (clitoroplasty, vaginoplasty, labioplasty); while older techniques were linked to reduced clitoral sensitivity and lower sexual satisfaction modern reconstructive approaches have evolved to prioritize neurovascular preservation and functional outcomes (36, 38, 99). Surgical techniques have significantly improved in recent years, and when performed in specialized centers, the risk of such complications has markedly decreased (100). Moreover, recent studies indicate that most women perceive the timing of their genital surgery as appropriate, and only a minority would opt for a different timing if given the choice, suggesting that contemporary surgical planning generally aligns with patients’ long-term expectations and satisfaction (101). The timing of genital surgeries remains a topic of ongoing debate, involving not only medical considerations but also ethical and human rights perspectives. Current discourse suggests that decisions regarding such procedures should be made collaboratively with parents and, when possible, with the patient herself. Ethical guidelines emphasize that, in general, once an adolescent reaches the age of 14, she has the right to provide sole consent to curative interventions, underscoring the importance of respecting the child’s developing autonomy (101, 102).

Studies have shown reduced quality of life among CAH patients, who tend to be single, less sexually active, less confident, less sociable, and feel less socially accepted (46, 103). Patients often have negative self-perception of their bodies, especially women with hyperandrogenism, who complain of menstrual dysfunction, hirsutism, and acne (38, 103). When comparing men and women, it is apparent that women have statistically significant lower scores in the WHOQOL-BREF questionnaire, which may be due to the ambiguity of genitalia and the need for genital surgery. Men, on the other hand, show statistically lower scores in the physical domain in the WHOQOL-BREF, since men tend to be diagnosed later. Females who underwent genital surgery showed statistically higher scores in the psychological domain compared to those who did not undergo surgery. The earlier the surgery was performed, the better quality of life was reported in adulthood (104, 105). Overall, the psychological impact of feminizing genital surgery in CAH appears heterogeneous: many studies report higher psychological-domain scores and satisfaction—particularly with earlier surgery performed in experienced centers—yet outcomes vary across cohorts.

Parents and patients considering genital reconstructive surgery should be fully informed about the risks of sexual function and the functional consequences of the disease, and the therapy should be explained by a female clinician experienced in this field to avoid concerns regarding sexual activities (106). While medical and surgical therapy seems to provide a near-satisfactory physical appearance of the genitalia in more than two-thirds of women with CAH, the frequency and significance of their reported sexual dysfunction and impaired reproductive outcomes highlight the need for advances in medical and surgical treatments (39).

**Figure 2:**
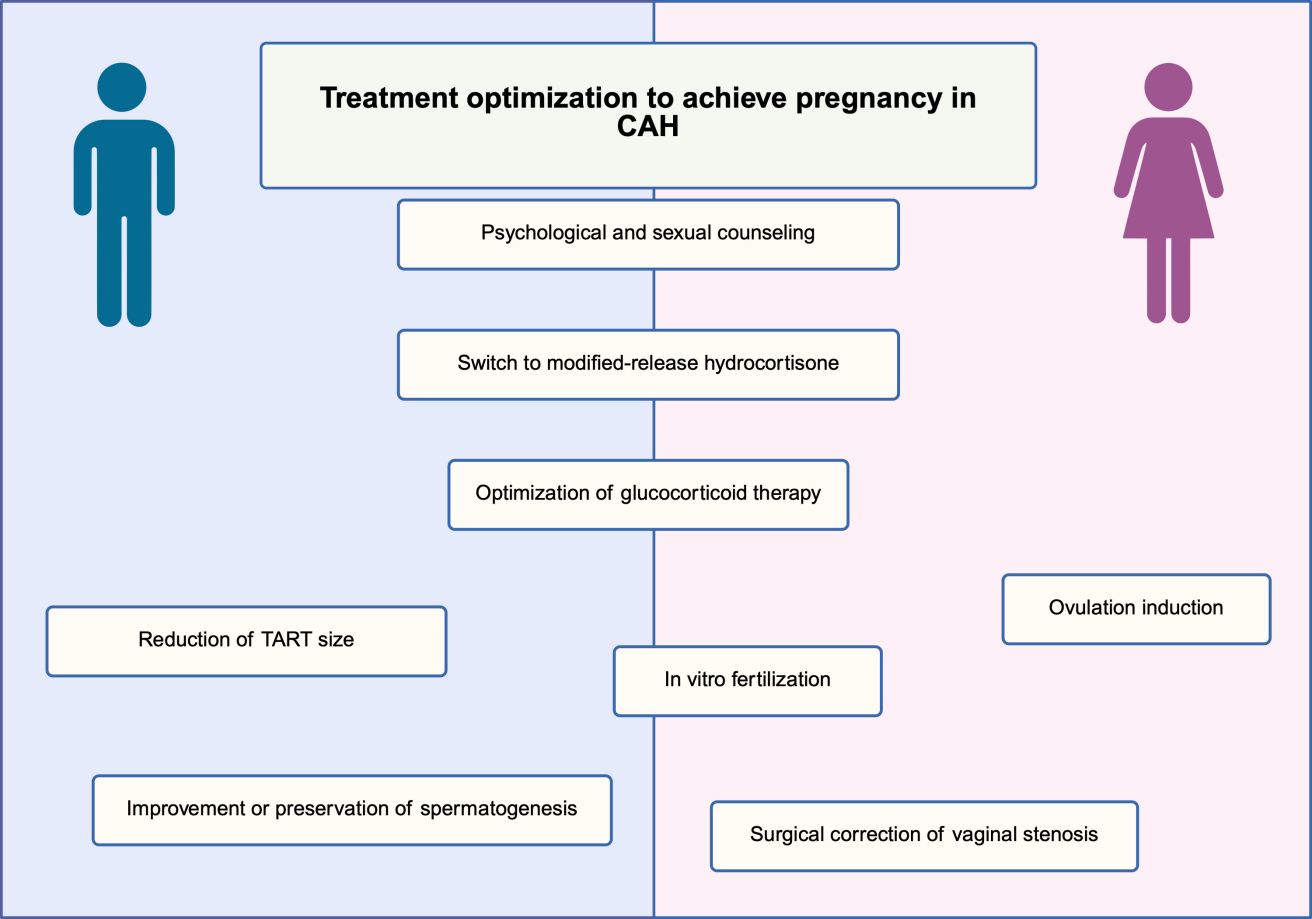
Treatment optimization strategies to achieve pregnancy in patients with congenital adrenal hyperplasia. Core interventions include switching to modified-release hydrocortisone, optimization of glucocorticoid therapy, and psychological and sexual counseling. Male-specific strategies focus on reduction of testicular adrenal rest tumor size and preservation of spermatogenesis. Female-specific strategies include ovulation induction, surgical correction of vaginal stenosis, and *in vitro* fertilization.

Proper education of CAH patients regarding their fertility, chances of becoming parents, or the risk of passing the condition to their offspring is also an important aspect. Misinformation can cause anxiety and discourage attempts to have children. In the dsd-LIFE study, up to 40% of 211 women with CAH did not remember receiving any information about their fertility potential, which highlights the need of complex care in these patients. Furthermore, of those who did received information, only 50% were satisfied with it, and only 60% were aware of the possibility of having their own biological children. These findings highlight the need for improved education of CAH patients about their fertility and their chances of passing the disease to their offspring (24, 107). The care of such patients should involve collaboration among specialists with good knowledge of the disease, including an endocrinologist, obstetrician, and psychologist. It is also important to provide genetic counselling and genetic testing of the partner, as further described below in this article (**Figure 2**).

## 6 Prenatal treatment

Once a woman with congenital adrenal hyperplasia (CAH) becomes pregnant, it is essential to assess the risk of CAH in the fetus. The most critical step in this process is performing genetic testing of the father to determine whether he is a carrier of mutations in the CYP21A2 gene, and this should be completed before attempting conception. The goal of early prenatal sex determination is to identify female fetuses who may be affected by the risk of virilization of the external genitalia due to excess endogenous fetal adrenal androgens in order to consider prenatal treatment with dexamethasone. Maternal androgens are inactivated by placental aromatase and do not contribute to fetal virilization (108). Dexamethasone crosses the placenta, where it suppresses fetal ACTH secretion and reduces adrenal androgen production; however, to be effective, treatment must be initiated before the 9th week of gestation, when differentiation of the external genitalia begins (51). While dexamethasone can suppress fetal adrenal glands and prevent virilization, it has potentially negative effects on brain development. Some studies have shown structural changes in the brain, such as amygdala enlargement and alterations in white matter (109, 110). However, results are conflicting, with some studies showing no adverse effects of prenatal dexamethasone therapy (111), while others report impacts on cognition, behavior, genome methylation, insulin secretion, and brain structures (55, 112). Long-term follow-up studies are still needed to fully understand the safety of this treatment (109, 110). It is also important to consider the adverse effects of excessive glucocorticoid use in the mother, which may include edema, hypertension, excessive weight gain, severe striae, sleep disturbances, Cushingoid features, irritability, gastrointestinal intolerance, and a hyperglycemic response to oral glucose administration in some cases (110).

Traditionally, dexamethasone has been administered at a dose of approximately 20 μg/kg/day (calculated based on pre-pregnancy maternal weight), divided into one to three daily doses, not exceeding 1.5 mg/day (113). However, more recent approaches, such as that proposed by Stachanow et al., suggest a significantly reduced regimen of 7.5 μg/kg/day due to the adverse events of the treatment (114). This low-dose strategy is currently being investigated in a prospective multicenter clinical trial (PREDICT study) led by Nicole Reisch, aiming to assess the efficacy and safety of reduced-dose prenatal dexamethasone in pregnancies at risk for CAH (115).

Prenatal diagnosis can be performed through chorionic villus sampling (CVS) at 9–11 weeks of pregnancy or amniocentesis at 15–20 weeks, followed by genetic testing. However, both procedures carry risks, with the fetal loss rates of 0.1% for amniocentesis and 0.2% for CVS (116). Recent advancements in prenatal testing have made it possible to determine fetal sex using Y-chromosome DNA found in maternal blood with an 99% accuracy (117). If Y-chromosome DNA is detected, indicating a male fetus, treatment is discontinued. If no Y-chromosome is found, further analysis of cell-free fetal DNA allows for genotyping of the CYP21A2 gene. If the fetus is confirmed to be both female and affected by 21-OHD, dexamethasone treatment may be continued throughout pregnancy to prevent virilization (51, 116).

By analyzing blood samples from both parents and an affected proband parallel DNA sequencing combined with a detailed analysis of single nucleotide polymorphisms near the CYP21A2 gene it is possible to successfully identify the CYP21A2 genotype in fetal DNA (118). However, this approach currently requires costly equipment and highly skilled personnel. Despite these limitations, it offers potential for improving the accuracy and speed of prenatal diagnosis in the years to come (3). The use of dexamethasone for prenatal treatment remains controversial. To prevent virilization of affected female fetuses, dexamethasone treatment is initiated empirically early in pregnancy—before the 9th week of gestation, when sexual differentiation begins. Treatment is discontinued if prenatal testing reveals an unaffected or male fetus, thereby avoiding unnecessary exposure to glucocorticoids.

## 7 Management during and after pregnancy

Pregnancy induces profound physiological changes in adrenal and gonadal steroidogenesis, which complicates biochemical monitoring. Placental production of progesterone and 17-OHP during pregnancy limits its usage as a marker of disease control. Moreover, androstenedione levels can increase by up to 80% by the end of the first trimester and remain elevated throughout gestation, further reducing their reliability (119). Assessing mineralocorticoid therapy based on plasma renin activity is problematic, as renin levels naturally rise during pregnancy and exhibits considerable interindividual variability (120, 121). That is why, adjustments should be based on clinical assessments, including blood pressure measurements, electrolyte balance, as well as clinical data. Trimester-specific steroid reference ranges are needed (51) and a few studies on gestational range gluco- and mineralocorticoids as well as androgens have already been published although they are not routinely used (122–124). Typically, pre-pregnancy GC and MC doses can be maintained during the first and second trimesters. Additionally, monitoring of testosterone levels is still conducted, but the specific thresholds may vary depending on the latest guidelines and clinical assessments (125). Recent studies suggest that from the third trimester onwards, a 20%-40% increase in GC dosage may be necessary to meet the higher cortisol requirements of the pregnant woman, although further research is needed to better define optimal dosing during this period (3, 126). In contrast, fludrocortisone doses typically remain unchanged unless clinical signs such as hypotension or hyperkalemia indicate the need for adjustment (51, 52). Underdosing GC risks hypoglycemia and adrenal crisis, while on the other hand overdosing may leads to unfavorable outcomes during pregnancy as described above (52). In case of adrenal crisis, urgent treatment with stress-doses of steroids and IV fluids is required (127), along with monitoring the fetus, especially after 24 weeks of pregnancy. The patient should be managed by an experienced endocrinologist and an obstetrician specializing in high-risk pregnancies (20, 128).

During labor, the dosage should be adjusted with 100 mg HCT i.v. at the start, followed by continuous infusion with 200 mg/24 h or 50 mg every 6 hours (52, 129). After uncomplicated labor, the hydrocortisone dose is reduced to 100 mg/day on day 1 (divided into 4 doses), 50 mg/day on day 2, with further reduction up to 35 mg/day on subsequent days (52). To appropriately adjust the doses, evaluation of clinical symptoms and signs of hypercortisolism or hypocortisolism is needed, in order to restore pre-pregnancy doses. What is more, early postpartum consultation with the endocrinology team is recommended (20). Women on cortisol replacement can breastfeed, as the glucocorticoid levels in breast milk are very low and unlikely affect development of the infant (130). Relative infant doses range from 0.35% to 0.53% for prednisone and 0.09% to 0.18% for prednisolone, and approximately 0.2%–0.5% for hydrocortisone (131). Current evidence indicates that no breastfeeding interruption is required following the intake of standard replacement doses of prednisone or prednisolone, unless higher pharmacological doses are used—such as in immunosuppressive therapy, in which recommendation states waiting 3 to 4 hours before breastfeeding to minimize infant exposure (131, 132).

## 8 Neonatal screening

CAH is a condition for which neonatal screening should be routinely performed within the first few days of life, due to its possible life-threatening outcome if not diagnosed and properly treated. Early detection through screening facilitates the timely initiation of treatment, accurate gender assessment in neonates with classic CAH, and helps prevent morbidity and mortality associated with adrenal crises which can be effectively managed with prompt administration of dexamethasone (8, 133). Screening is conducted in most European countries, except Albania, and is also implemented across all U.S. states, as well as in many countries in South America and Asia (134).

The screening involves collecting a small blood sample from the infant’s heel (typically between the second and fourth day of life), which is then dried on filter paper and analyzed for 17-OHP levels using a fluorometric assay. When elevated levels (over 50 ng/dl for newborns with weight >2500 g) are found, follow-up testing is performed. This includes a more detailed steroid profile using liquid chromatography–tandem mass spectrometry (LC-MS/MS), which measures additional markers such as cortisol, 21-deoxycortisol, 11-deoxycortisol, and androstenedione (135).

Evidence increasingly supports that early diagnosis through screening can be lifesaving—particularly for male newborns. Unlike females with classic CAH, who often present with ambiguous genitalia and are diagnosed more readily, affected males may initially appear physically typical, leading to delayed recognition of a potentially life-threatening salt-wasting crisis (136). Historically, the low number of diagnosed males with CAH raised concerns about undiagnosed cases potentially leading to fatal outcomes before proper treatment could be administered. However, with the introduction of newborn screening programs, the early detection of CAH, including in males, has significantly reduced the risk of death due to adrenal crises and has improved the prognosis for affected infants. In fact, retrospective studies from regions without universal screening have consistently reported a higher prevalence of CAH among females, hinting at possible underdiagnosis—or even fatal outcomes—among boys (136, 137). Moreover, data suggest that newborns identified through screening programs are more likely to carry severe genetic mutations compared to those diagnosed clinically prior to the implementation of screening (137). This observation may reflect a survival bias, where only those with milder forms of the disease survived long enough to be diagnosed before screening was available.

## 9 Conclusions

Fertility issues in 21-OHD affect both women and men, but with appropriate management, fecundity comparable to the general population can be achieved. In males, impaired fertility may result from TARTs, suppressed gonadotropin secretion, suboptimal hormonal control, and genotype-related differences in residual enzyme activity and adrenal androgen production (85, 86, 94). In females, it can be improved by multiple factors, including phenotype and mutation type — both determining residual enzymatic activity and androgen levels — as well as advances in surgical techniques for genital reconstruction, early and effective medical management, and improved treatment adherence (21, 37, 46, 68, 106). Access to psychological support is important, as emotional well-being strongly influences reproductive health. A well-structured transition from pediatric to adult endocrine care is also essential, as continuity of specialized management can optimize long-term outcomes (8).

Furthermore, effective regulation of the menstrual cycle and the promotion of sexual health are key components in improving both fertility rates and overall quality of life in women with CAH. Moreover, due to relatively high prevalence of carriers in the general population, CAH patients who inherit two severe mutations in the *CYP21A2* gene, should encourage their partners for, genetic screening, which is of paramount importance (138). Identifying a carrier partner refines the risk of having a child with classic CAH and supports informed reproductive choices; genetic counseling should be readily available to guide family planning and potential fertility treatments. Psychological distress is common, particularly among women, due to factors such as hirsutism, menstrual irregularities, and body image concerns. While surgical interventions may enhance psychological well-being, their long-term effects remain debated. Prenatal diagnosis and dexamethasone treatment aim to reduce virilization in female fetuses, but concerns persist regarding potential neurodevelopmental effects (139). Postnatal management requires individualized hormone therapy and monitoring, particularly during pregnancy (107, 131). Newborn screening and genetic counseling play a crucial role in early detection and family planning (133). Further research is needed to optimize surgical techniques, improve long-term quality of life, and refine prenatal treatment strategies.
